# Bi-allelic Mutations in Phe-tRNA Synthetase Associated with a Multi-system Pulmonary Disease Support Non-translational Function

**DOI:** 10.1016/j.ajhg.2018.06.006

**Published:** 2018-07-05

**Authors:** Zhiwen Xu, Wing-Sze Lo, David B. Beck, Luise A. Schuch, Monika Oláhová, Robert Kopajtich, Yeeting E. Chong, Charlotte L. Alston, Elias Seidl, Liting Zhai, Ching-Fun Lau, Donna Timchak, Charles A. LeDuc, Alain C. Borczuk, Andrew F. Teich, Jane Juusola, Christina Sofeso, Christoph Müller, Germaine Pierre, Tom Hilliard, Peter D. Turnpenny, Matias Wagner, Matthias Kappler, Frank Brasch, John Paul Bouffard, Leslie A. Nangle, Xiang-Lei Yang, Mingjie Zhang, Robert W. Taylor, Holger Prokisch, Matthias Griese, Wendy K. Chung, Paul Schimmel

**Affiliations:** 1 IAS HKUST - Scripps R&D Laboratory, Institute for Advanced Study, Hong Kong University of Science and Technology, Clear Water Bay, Kowloon, Hong Kong, China; 2 Pangu Biopharma, Edinburgh Tower, The Landmark, 15 Queen’s Road Central, Hong Kong, China; 3 aTyr Pharma, 3545 John Hopkins Court, Suite 250, San Diego, CA 92121, USA; 4 Department of Medicine, Columbia University, New York, NY 10032, USA; 5 National Human Genome Research Institute, National Institutes of Health, Bethesda, MD 20892, USA; 6 Dr. von Hauner Children’s Hospital, Division of Pediatric Pneumology, University Hospital Munich, German Center for Lung Research (DZL), Lindwurmstr. 4, 80337 München, Germany; 7 Wellcome Centre for Mitochondrial Research, Institute of Neuroscience, The Medical School, Newcastle University, Newcastle upon Tyne NE2 4HH, UK; 8 Institute of Human Genetics, Technical University Munich, 81675 Munich, Germany; 9 Institute of Human Genetics, Helmholtz Zentrum München, Deutsches Forschungszentrum für Gesundheit und Umwelt (GmbH), Ingolstädter Landstr. 1, 85764 Neuherberg, Germany; 10 Department of Pediatrics, Columbia University, New York, NY 10032, USA; 11 Goryeb Children’s Hospital, Atlantic Health System, Morristown, NJ 07960, USA; 12 Department of Pathology, Weill Cornell Medicine, New York, NY 10065, USA; 13 Department of Pathology and Cell Biology, Columbia University, New York, NY 10032, USA; 14 Taub Institute for Research on Alzheimer’s Disease and the Aging Brain, Columbia University, New York, NY 10032, USA; 15 GeneDx, Gaithersburg, MD 20877, USA; 16 Center for Human Genetics and Laboratory Diagnostics (AHC) Dr. Klein, Dr. Rost and Colleagues, Lochhamer Str. 29, 82152 Martinsried, Germany; 17 Department of Pediatrics and Adolescent Medicine, University Medical Center, Medical Faculty, University of Freiburg, 79085 Freiburg, Germany; 18 Bristol Royal Hospital for Children, University Hospitals Bristol NHS Foundation Trust, Bristol BS2 8BJ, UK; 19 Royal Devon & Exeter NHS Foundation Trust, Exeter EX2 5DW, UK; 20 Institut für Neurogenomik, Helmholtz Zentrum München, Deutsches Forschungszentrum für Gesundheit und Umwelt (GmbH), Ingolstädter Landstr. 1, 85764 Neuherberg, Germany; 21 Klinikum Bielefeld Mitte, Institute for Pathology, Teutoburger Straße 50, 33604 Bielefeld, Germany; 22 Department Pathology, Morristown Memorial Hospital, Morristown, NJ 07960, USA; 23 The Scripps Laboratories for tRNA Synthetase Research, The Scripps Research Institute, 10650 North Torrey Pines Road, La Jolla, CA 92037, USA; 24 Department of Molecular Medicine, The Scripps Research Insitute, La Jolla, CA 92037, USA; 25 Division of Life Science, State Key Laboratory of Molecular Neuroscience, Hong Kong University of Science and Technology, Clear Water Bay, Kowloon, Hong Kong, China; 26 The Scripps Laboratories for tRNA Synthetase Research, Scripps Florida, 130 Scripps Way, Jupiter, FL 33458, USA

**Keywords:** tRNA synthetase, exome sequencing, bi-allelic mutation, non-coding variant, missense mutation, multi-system disease, cerebral calcifications, cholesterol pneumonitis, interstitial lung disease, non-canonical function

## Abstract

The tRNA synthetases catalyze the first step of protein synthesis and have increasingly been studied for their nuclear and extra-cellular ex-translational activities. Human genetic conditions such as Charcot-Marie-Tooth have been attributed to dominant gain-of-function mutations in some tRNA synthetases. Unlike dominantly inherited gain-of-function mutations, recessive loss-of-function mutations can potentially elucidate ex-translational activities. We present here five individuals from four families with a multi-system disease associated with bi-allelic mutations in *FARSB* that encodes the beta chain of the alpha_2_beta_2_ phenylalanine-tRNA synthetase (FARS). Collectively, the mutant alleles encompass a 5′-splice junction non-coding variant (SJV) and six missense variants, one of which is shared by unrelated individuals. The clinical condition is characterized by interstitial lung disease, cerebral aneurysms and brain calcifications, and cirrhosis. For the SJV, we confirmed exon skipping leading to a frameshift associated with noncatalytic activity. While the bi-allelic combination of the SJV with a p.Arg305Gln missense mutation in two individuals led to severe disease, cells from neither the asymptomatic heterozygous carriers nor the compound heterozygous affected individual had any defect in protein synthesis. These results support a disease mechanism independent of tRNA synthetase activities in protein translation and suggest that this FARS activity is essential for normal function in multiple organs.

## Introduction

The universal aminoacyl tRNA synthetase (aaRS) family of enzymes is necessary for protein synthesis and is increasingly implicated in key signaling pathways outside of protein synthesis. In that connection, they have been associated with previously unrecognized human diseases and functions.^1^, ^2^, ^3^, ^4^, ^5^ aaRSs have nuclear and extra-cellular activities that in some cases integrate translation with cell signaling pathways as well as functions independent of protein translation. The evolution of these novel functions correlates with the addition of novel domains, such as nuclear localization signals or receptor-binding motifs, which are not essential for aminoacylation.^6^, ^7^, ^8^, ^9^, ^10^, ^11^, ^12^, ^13^ Further highlighting the polybiology of human aaRSs is the large number (about 250) of splice variants, the majority of which ablate or disrupt the catalytic domain necessary for protein translation and yet retain novel elements that likely are not directly involved in protein synthesis.^14^

Mutations in cytoplasmic and mitochondrial aaRSs in humans are associated with neuropathies, leukoencephalopathies, myopathies, hepatopathies, and lung disorders.^15^, ^16^, ^17^, ^18^ Dominant missense mutations in aaRSs associated with Charcot-Marie-Tooth disease (CMTD) are the most common, and in some cases have been studied in mechanistic detail.^19^ More than a dozen different CMTD mutations have been described in glycyl-tRNA synthetase (GARS).^20^ For some mutations, an extracellular form of mutant GARS binds neuropilin-1 to block binding of vascular endothelial growth factor A to its receptor and interferes with a critical neuronal signaling pathway.^21^, ^22^ While these dominant gain-of-function mutations are informative, recessive aminoacylation-disruptive mutations in aaRSs might be most useful for elucidating a novel function of aaRSs beyond protein translation.

The phenotypes associated with recessive mutations emerge because cytoplasmic and mitochondrial genes for aaRSs are single copy and, with rare exception, there are distinct genes for the cytoplasmic and mitochondrial forms. Bi-allelic mutations in genes for lysyl, glycyl, methionyl-, isoleucyl-, glutamyl-, and arginyl-tRNA synthetases have been reported.^23^, ^24^, ^25^, ^26^, ^27^, ^28^, ^29^, ^30^ Phenotypes associated with these mutations are variable and whether they arise from defects in protein synthesis has either not been explored or is controversial. However, mice haploinsufficient for GARS have a 50% decrease in enzymatic activity *in vitro* and in tissues, but nonetheless have a normal phenotype and lifespan.^31^ Thus, if protein synthesis is not dramatically affected, humans with one loss-of-function allele or retaining some residual activity might be viable.

Here, we report five individuals from four independent families with bi-allelic mutations in the gene for the beta chain of *FARS* (MIM: 609690) associated with a complex, unique phenotype associated with cholesterol pneumonitis, cerebral aneurysms and calcifications, and liver cirrhosis. The associated phenotype can be lethal and is more severe than that observed with most other recessive aaRS diseases reported to date. In our study, we investigated one family in detail and demonstrate no impact of these mutations on protein synthesis in cells. These data are thus consistent with the bi-allelic mutations causing a change in one or more *FARSB* functions outside of protein synthesis. These functions appear to be essential for multiple organ function.

## Material and Methods

Informed consent was obtained from all participants included in the study. The Institutional Review Board of Columbia University, the National Research Ethics Service Committee North East – Newcastle & North Tyneside 1, and the Ethics Committee of the University of Munich, Germany^32^ approved this study. All of the procedures followed were in accordance with the ethical standards of each institution.

### DNA Extraction and Exome Sequencing

Genomic DNA was extracted from whole blood from proband 1, his parents, and autopsy materials from his deceased sister. Given the unique constellation of signs and symptoms and the familial nature, exome sequencing of both parents, both affected siblings, and a third unaffected older female sibling was performed as previously described.^33^ Exome sequencing was performed on exon targets captured using the Agilent SureSelect Human All Exon V4 (50 Mb) kit. After automated filtering of variants with a minor allele frequency (MAF) of >10%, manual curation was performed to filter less common variants with MAF of 1%–10% and variants in genes inherited from unaffected parents, evaluating predicted effects of rare variants and known function of the genes and associated human conditions, and examining overlapping phenotypes of individuals with *de novo* variants in the same gene as previously described.^33^ In addition to allele frequency, pathogenicity prediction algorithms, such as PolyPhen2,^34^ SIFT,^35^ CADD,^36^ REVEL,^37^ and MetaSVM,^38^ were used for prioritizing variants.

Genomic DNA from participants 3, 4, and 5 and their parents was isolated from whole blood using the chemagic DNA Blood Kit special (PerkinElmer) or the QIAmp DNA Mini Kit (QIAGEN), according to the manufacturer’s protocol. Exome sequencing was performed as previously described.^39^ Exonic regions were enriched using the SureSelect Human All Exon kit (50Mb_v5) from Agilent followed by sequencing as 100 bp paired-end runs on an Illumina HiSeq2500. Exome sequencing for participants 3 and 4 and their parents was performed by Personalis (Personalis). Reads were aligned to the human reference genome (UCSC Genome Browser build hg19) using Burrows-Wheeler Aligner (v.0.7.5a). Identification of single-nucleotide variants and small insertions and deletions (indels) was performed with SAMtools (v.0.1.19). For analysis of rare bi-allelic variants, only variants with a minor allele frequency (MAF) of less than 1% in our internal database of 14,000 exomes were considered.

### Tissue Immunohistochemistry, Cell Preparation, and Culture

Tissue preparation, immunostaining, and semiquantitative evaluation were done as described.^40^ Lymphoblastoid cell lines were maintained after EBV immortalization. Fibroblasts were cultured from a 3 mm punch skin biopsy. In addition to these cells, we also purchased a control immortalized PBMC (ATCC #CRL5959) of a normal individual (a 58-year-old male, European descent) and two control primary fibroblasts (Coriell #GM07753 and #GM07492) of apparently healthy individuals (both are 17-year-old males, European descent, named CTL-17Ma and -17Mb, respectively). All the immortalized PBMCs were maintained in Iscove’s Modified Dulbecco’s Medium (IMDM) supplemented with 15% fetal bovine serum (FBS) and 4 mM L-Glutamine (Thermo Scientific). For prevention against bacteria, fungi, and mycoplasma, the media also contained Penn Strep (Thermo Scientific), Normocin (Invivogen), Nystatin suspension, and Amphotericin B solution (Sigma Aldrich) according to manufacturers’ instructions. The primary fibroblasts were maintained in Dulbecco’s Modified Eagle’s medium (DMEM) containing high glucose and GlutaMAX (Thermo Scientific) and supplemented with 15% FBS and 1% Penn Strep. Cells were incubated at 37°C in a humidified CO_2_ incubator. The immortalized PBMCs of less than 30 passages and primary fibroblasts of less than 20 passages were employed in the experiments.

### Modeling of the Human FARS-tRNA^phe^ Complex Structure

The structure of the *Thermus thermophilus* tRNA^Phe^ molecule (PDB: 2IY5) was docked into the crystal structure of human FARS (PDB: 3L4G) using the PathDock server^41^ and manually adjusted as previously described.^42^ All the structural figures were prepared with Pymol.

### RNA Isolation and Quantitative Real-Time PCR

Cultured cells were washed with 1× PBS twice before RNA extraction. Total RNA was extracted using RNAeasy Mini kit (Life Technologies) with on-column TURBO DNase digestion (Life Technologies). Isolated total RNA was quantified by NanoDrop 1000 spectrometer. For gene expression assessment by quantitative real-time PCR, messenger RNA was captured by oligo(dT)18 primer and reverse-transcribed into cDNA using SuperScript III Reverse-Transcriptase (Life Technologies). To minimize variation, 2 μg of total RNA was input in each reverse transcription reaction. The cDNA then was purified using DNA purification kit (Omega) and eluted in 400 μL of sterile water. The quantitative real-time PCR was performed as described previously.^14^ The expression of target genes was normalized to that of the housekeeping genes *RPL9* and *RPL11* in each sample.

### Western Blotting and Relative Band Intensity Analysis

Frozen aliquots of cells were lysed by the radioimmunoprecipitation assay (RIPA) buffer (Bio-Rad) added with complete protease inhibitors (Roche) at 4°C for 15 min. The crude lysates were further sonicated and followed with centrifugation at 12,000 × *g* for 15 min at 4°C. The supernatant containing total cell extracts was quantified for protein concentration by the Pierce BCA Protein Assay (Thermo Scientific). The western blot was performed using primary antibodies (a-FARSb: Abnova #H00010056-M01; a-FARSa: Abcam #54653) and the HRP-conjugated secondary antibody. Protein bands were visualized by chemiluminescence on the ChemiDoc Imaging System (Bio-Rad). The relative band intensity was quantified by the Image Lab software (Bio-Rad) according to manufacturer’s instructions.

### Aminoacylation Assay

Whole-cell extracts of immortalized PBMCs were prepared by lysis with M-PER Mammalian Protein Extraction Reagent (Thermo Scientific) in the presence of Halt Protease Inhibitor (Thermo Scientific). Crude lysates were centrifuged at 14,000 × *g* for 10 min at 4°C. The supernatant containing total cell extracts was transferred to clean eppendorf tubes. Protein concentrations were measured using the Quick Start Bradford Protein Assay (Bio-Rad). An equal amount of protein (7.5 μg per reaction) from each sample was added to reaction mix containing 12.5 mg/mL yeast tRNA (Sigma-Aldrich), 300 μM L-Phe or L-Gly, 1.6 μM [^3^H]-Phe (American Radiolabeled Chemicals) or 6 μM [^3^H]-Gly (Perkin Elmer), 4 mM ATP, 10 mM MgCl_2_, 2 mM DTT, 20 mM KCl, and 50 mM HEPES (pH 7.5). The reaction was stopped by addition of quench solution consisting of 0.5 mg/mL DNA, 100 mM EDTA, and 300 mM NaOAc (pH 3.0). [^3^H]-Phe- or [^3^H]-Gly-labeled tRNA was precipitated by addition of 20% TCA and captured using Multiscreen HTS filter plates (EMD Millipore). Filters were washed with a 5% TCA, 100 mM Phe solution and captured tRNA was solubilized with 0.1 M NaOH. Amount of [^3^H] was quantified by addition of Optiphase Supermix scintillation cocktail (Perkin Elmer) and counting by a MicroBeta scintillation counter (Perkin Elmer).

### Puromycin Incorporation Assay

Puromycin is a structural analog of tyrosyl-tRNA and can incorporate into nascent polypeptide chains during protein translation. Its incorporation in cultured cells followed with detection using anti-puromycin antibodies has been developed as a well-validated method to reflect the rate of global protein synthesis.^43^, ^44^ The cells were seeded 1 day before treatment and incubated overnight. The seeding density was 6K cells/96-well for immortalized PBMCs and 30K cells per 24-well for primary fibroblasts. The next day, after media refresh, cells were pulsed with or without puromycin for 10 min. For cycloheximide (CHX) inhibition of protein synthesis, cells were pre-treated with 400 μg/mL CHX (Sigma) for 30 min. Media were refreshed after the puromycin ± CHX treatment and cells were incubated in clean media for 1 hr in the CO_2_ incubator. Then, cells were washed and detached with Accutase cell detachment solution (Thermo Scientific) and transferred to the 96-well V-bottom plate for staining. The immortalized PBMCs from 4 × 96-wells were combined and fibroblasts from 1 × 24-wells were directly transferred to 1 staining well. After PBS wash and spin down at 300 × *g* for 5 min, cells were first stained with 100 μL live/dead Zombie NIR (Biolegend) at 1:1,000 dilution for 15 min at room temperature. After PBS wash, the cells were resuspended in 60 μL Cytofix/Cytoperm solution (BD Biosciences) and incubated on ice for 20 min. Cells were washed twice with Perm/Wash buffer (BD Biosciences) and incubated with a-puromycin (DHSB #PMY-2A4) for 45 min on ice in the dark. After washing, cells were incubated with a-mouse IgG NL493 (R&D Systems) for 45 min, and finally resuspended in FACS buffer (1× PBS supplemented with 2% FBS) and kept at 4°C until flow cytometry analysis by the FACSARIA III system (BD Biosciences). The median fluorescence intensity (MFI) for each puromycin dose minus that of the blank (puromycin = 0) was employed for curve fitting by Prism (Graphpad) using the log(agonist) versus response − variable slope nonlinear regression to calculate EC_50_ of the puromycin incorporation.

### Cell Proliferation Assay

Cell growth was determined using CellTiter-Glo Luminescent Cell Viability Assay reagent (Promega) following manufacturer’s instructions. Fibroblast cells were plated on 96-well plates at 1,000 cells per well and cultured in complete medium. Proliferation was measured every 24 hr for 120 hr and therefore at six time points in total. Cells were lysed using freshly prepared assay reagent and incubated for 10 min at each end point. Cell lysates were then transferred to white flat-bottom plate for luminescence measurement on a FLOUstar Optima plate reader (BMG labtech). Cell growth was calculated relative to time 0 for each cell.

### Statistical Analysis

Results of at least three biological replicates were represented as mean ± standard error of the mean (SEM). The significance of difference between two groups was analyzed by Student’s t test, and among multiple groups was analyzed by the one-way analysis of variance (ANOVA) followed with Newman-Keuls’ multiple comparison tests. The p values of less than 0.05 were regarded as statistically different.

## Results

### Clinical Characteristics of Participants

**Figure 1:**
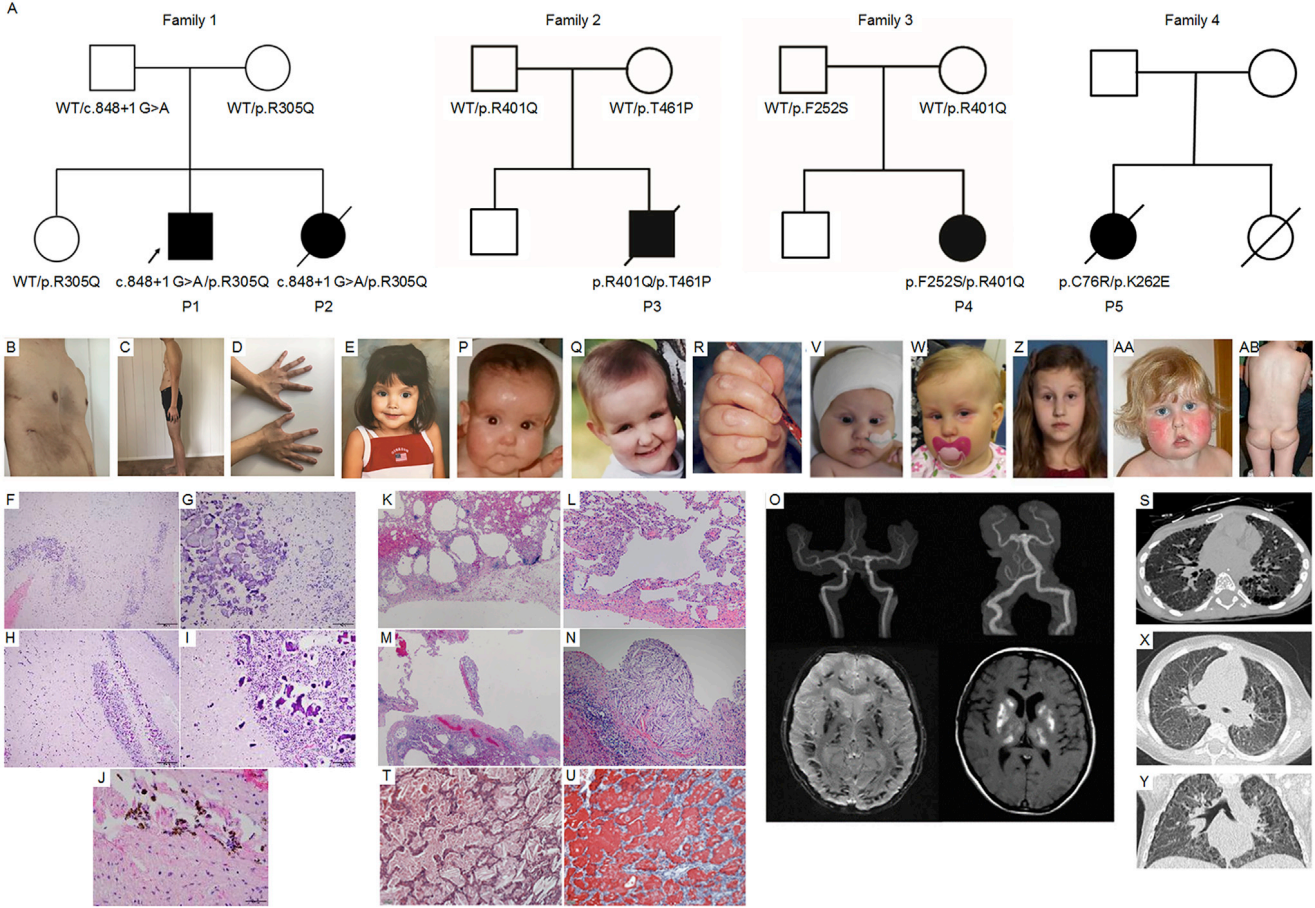
Segregation of *FARSB* Mutations in the Family and Clinical Findings in the Subjects (A) Pedigree with *FARSB* genotypes listed below each family member. (B–D) Images of proband P1 highlighting pectus excavatum, poor wound healing, and arachnodactyly. (E) Image of proband P2. (F–J) Clinical findings in the proband P2. Low (F)- and high (G)-power views of the cortex. Low (H)- and high (I)-power views of the cerebellum. Mineral deposits, consistent with calcifications (G–I). Hemosiderin-laden macrophages in cortex (J), consistent with prior bleeding. (K–O) Clinical findings in the proband P1. Histopathology of lung revealing emphysema, sub-pleural fibrosis, and cholesterol granuloma. (K) Lung tissue shows diffuse alveolar simplification with dilated alveolar spaces. (L) Emphysema is present with alveolar duct dilatation and mild interstitial thickening around dilated airspaces. (M) Areas of severe bullous emphysema with characteristic floating “broken” septa are observed. (N) Patches of macrophage accumulation and cholesterol clefts are observed, indicative of prior tissue injury. These areas also had dystrophic calcification (not shown). (O) Brain imaging showing multiple large aneurysms and cerebral calcifications. (Top left) Magnetic resonance angiogram of the brain without contrast showing the anterior circulation with approximately 3 mm right internal carotid artery cavernous aneurysm and a large fusiform dilatation of the left carotid terminus extending into the left anterior carotid artery and left middle carotid artery. (Top right) Similar aneurysms present in posterior circulation including a large, irregular fusiform aneurysm involving the basilar tip, right superior cerebral artery, and right posterior cerebral artery, and to lesser extent left posterior cerebral artery. (Bottom left) MRI brain without contrast, susceptibility weighted imaging signal with low signal throughout the bilateral subcortical white matter within the cerebellar and cerebral hemispheres. These findings again likely represent calcifications. (Bottom right) T1 shortening embolism within the bilateral deep gray nuclei likely representing calcifications, less likely hemorrhage. (P–R) Images of proband P3 were taken at age 0.5 (P), 5 (Q), and 6 (R) years. Note frontal bossing, deep and narrow-set eyes (P, Q), and pads on dorsal proximal phalanges (R). (S–U) Clinical findings in P3. Chest imaging showed interstitial lung disease with diffuse ground glass attenuation and subpleural and paraseptal cystic lesions (S). Lung histology with intraalveolar and interstitial cholesterol granuloma with giant cells and cholesterol crystals, round cell infiltrate, foamy macrophages, cuboid metaplasia of alveolar type II cells, subpleural emphysematous spaces, increased intraalveolar accumulation of surfactant (T, HE-stain), also demonstrated by strong staining with antibodies against SP-A (U), summarized as focal cholesterol pneumonitis and pulmonary alveolar proteinosis. (V–Z) Images of proband P4 were taken at age 4 months (V), age 16 months (W), and age 10 years (Z). Note prominent forehead and narrow-set eyes (V, W). Initial CT with bilateral interstitial consolidations and ground-glass opacity in the upper lobes, more on the left than right, and bilateral pleural effusion. A year later, previously affected areas are cystic, as well as subpleural cystic lesions, interlobular septal thickening, and ground-glass attenuation (X, Y). (AA and AB) Images of proband 5 were taken at 4 years old and demonstrate prominent forehead, full cheeks, deep-set eyes, myopathic facies (AA), and unusual fat distribution (AB).

The clinical features of the five individuals are summarized in Table 1. We evaluated a family with two similarly affected children with recurrent pneumothoraces, interstitial lung disease, hypertension, intracranial aneurysms and calcifications, and cirrhosis of unclear etiology. Participant 1 (P1, Figures 1A–1D) is the second child born to unaffected, non-consanguineous parents (father of Irish/English descent and mother of Filipino descent). The pregnancy was uncomplicated, and birth parameters were within normal limits. He had a history of congenital hypothyroidism. The proband presented at 6 months of age with failure to thrive requiring gastrostomy tube placement. Further neurologic workup revealed diffuse hypotonia and decreased muscle mass, with a muscle biopsy showing hypereosinophilia of myocytes with diminished type 1 muscle fibers and normal mitochondrial oxidative phosphorylation. He subsequently had delayed motor milestones, first walking at age 3, without any evidence of intellectual disability. He had a spontaneous pneumothorax at the age of 13 and imaging and histological evaluation of the lungs demonstrated pulmonary blebs, severe emphysematous changes, fibrosis, and cholesterol granuloma consistent with prior injury (Figures 1K–1N). He had evidence of restrictive lung disease and decreased lung volumes on pulmonary function testing. He developed hypertension at the age of 14, and angiography identified stenosis of the distal right renal artery. Pathological examination of the renal artery showed marked disruption of the internal elastic lamina and fibrosis consistent with fibromuscular dysplasia. Vascular imaging demonstrated extensive intracerebral aneurysms within the anterior and posterior circulation and fusiform dilatations of the right carotid artery and basilar artery (Figure 1O). Brain MRI showed symmetric calcifications of the subcortical white matter in the cerebellum and cerebrum (Figure 1O). Notably, all echocardiograms including aortic dimensions were normal. As an adolescent, he developed scoliosis and pectus excavatum. He has a history of low bone mineral density and poor wound healing. Upon last evaluation at the age of 18 years, he was 170 cm tall and 51.2 kg (BMI 17.7 kg/m^2^) with a physical examination notable for features that overlap with Marfan syndrome including micrognathia, tooth crowding, arachnodactyly, positive wrist and thumb signs, joint hyperextensibility, scoliosis, pectus excavatum, and abdominal hernia (Figures 1B–1D). He has no evidence of cognitive deficits and attends college.

**Table 1:** Clinical Features of the Five Participants

**Participant**	**Family**	**Gender**	**Status**	***FARSB* Genotype (NM_005687.4)**	**Minor Allele Frequency (gnomAD)**	**CADD Score****^36^**	**Lung Histopathology**	**Brain MRI Findings**	**Brain Angiography**	**Connective Tissue Findings**	**Liver**	**Kidney**	**Hypertension**	**Muscle Hypotonia**	**Intestinal Malrotation**	**Dysmorphic Features**
1	1	M	18 yo	c.848+1G>A (p.Arg305Gln)	.001%, .0008%	28.9, 35	pulmonary blebs, severe emphysematous changes, fibrosis, and cholesterol accumulation	symmetric calcifications of the subcortical white matter in the cerebellum and cerebrum	extensive intracerebral aneurysms within the anterior and posterior circulation, and fusiform dilatations of the right carotid artery and basilar artery	scoliosis, pectus excavatum, poor wound healing, arachnodactyly, positive wrist and thumb signs, joint hyperextensibility, low bone mineral density, and abdominal hernia	cirrhosis	renal artery stenosis	with renal artery stenosis	+	none	micrognathia, tooth crowding
2	1	F	deceased (10 yo)	c.848+1G>A (p.Arg305Gln)	.001%, .0008%	28.9, 35	bilateral pulmonary fibrosis with severe chronic interstitial pneumonitis and cholesterol crystals	diffuse calcifications and encephalomalacia	extensive intracranial aneurysms	scoliosis	cirrhosis	none	untreated but likely responsible for brain aneurysm hemorrhage	+	+	none
3	2	M	deceased (8 yo)	p.Arg401Gln, p.Thr461Pro	not present, not present	34, 31	interstitial lung disease with fibrosis, alveolar and interstitial accumulation of cholesterol granulomas, cysts, pulmonary alveolar proteinosis	leukoencephalopathy with symmetric calcifications in basal ganglia and cerebellum, hydrocephalus e vacuo	not assessed	pectus excavatum, low bone mineral density, hyperextensibility of joints	not assessed, chronically elevated liver enzymes	mild incomplete tubular proteinuria		+	+	frontal bossing, deep and narrow-set eyes
4	3	F	10 yo	p.Phe252Ser, p.Arg401Gln	not present, not present	29.9, 34	interstitial lung disease with cholesterol pneumonitis, non-specific interstitial pneumonitis, desquamative interstitial lung disease, bronchial wall thickening, cysts, formation of giant cells phagocytosing hemosiderin, cholesterol crystals	not assessed	not assessed	none	increased liver size, elevated liver enzymes first 4 y of life, later normal	severe bilateral vesico-urethral reflux (grade 3-4), treated by operation		+	none	prominent forehead, narrow-set eyes
5	4	F	deceased (8 yo 8 m)	p.Cys76Arg, p.Lys262Glu	not present, not present	27.2, 27.1	severe interstitial pneumonitis, lymphocyte and plasma cell infiltrate, cholesterol clefts, multinucleate giant cells, fibrosis and cysts	no significant abnormality at 4 months	not assessed	none	moderate steatosis	focal segmental glomerulo-sclerosis	pulmonary hypertension	+	none	prominent forehead, full cheeks, small nose, narrow deep-set eyes, myopathic facies, unusual fat distribution over buttocks

The proband’s younger sister (P2, Figures 1A and 1E) had similar clinical features including difficulty gaining weight, hypotonia, early gross motor delays, marfanoid body habitus, scoliosis, recurrent pneumothoraces, cirrhosis, intracranial aneurysms, stroke, hypertension, and seizures. She died at 10 years of age due to an intracranial hemorrhage caused by a ruptured aneurysm. Her autopsy showed bilateral pulmonary fibrosis with severe chronic interstitial pneumonitis and cholesterol crystals. She had hepatomegaly with early cirrhosis, malrotation of the intestines, and skeletal muscle myofiber enlargement and hypereosinophilia. Within the heart there were scattered hypereosinophilic myocytes. Her brain was microcephalic with diffuse calcification in the cerebellum and cerebrum, encephalomalacia, and multiple saccular aneurysms (Figures 1F–1J).

Participant 3 (P3, Figures 1A and 1P–1R) was the second child born to unaffected, non-consanguineous parents of European descent. The pregnancy, birth, and neonatal period were uncomplicated. At the age of 3 months, he had recurrent vomiting, was noted to be hypotonic, and was incidentally found to have intestinal malrotation. A brain MRI at 1 year of age showed demyelination of the nucleus lentiformis and corona radiata. Muscle biopsy demonstrated structurally normal but hypotrophic skeletal muscle with increased fat accumulation and biochemical analysis was suspicious of a partially impaired cytochrome-c-oxidase (complex IV) activity. The individual had low bone mineral density, mild tubular proteinuria, frontal bossing, and deep-set eyes and had psychomotor delay and speech delay before entering school (Figures 1P–1R). An MRI at the age of 6 years showed dilation of the lateral ventricles and leukoencephalopathy with symmetric calcifications in the basal ganglia and cerebellum. He was later hospitalized with clubbing, pectus excavatum, inspiratory wheezing, tachydyspnea, and retractions. Chest imaging demonstrated bilateral diffuse ground glass, increased reticular interstitial markings, and cysts (Figure 1S). Histologic evaluation of the lungs demonstrated alveolar and interstitial accumulation of huge cholesterol granulomas with foreign-body giant cells, consistent with cholesterol pneumonia in combination with extensive areas of pulmonary alveolar proteinosis (Figure 1T), as demonstrated by alveolar filling with surfactant protein (SP) A-positive material (Figure 1U). Additional staining demonstrated the presence of SP-B, pro-SP-B, but not pro-SP-C and SP-D, and alveolar macrophages and T lymphocytes (Figures S1A–S1C). Due to respiratory failure, he received a lung transplant at the age of 8. The explanted lungs were grossly yellow and stiff, confirming severe cholesterol pneumonitis and fibrosis. The post transplantation course was complicated with chronic effusion and infections, and he died 10 months later due to transplant rejection.

Participant 4 (P4, Figures 1A, 1V, 1W, and 1Z) is the second child of four children born to healthy, non-consanguineous parents of European ancestry. Distinct facial features included a prominent forehead and narrow-set eyes (Figures 1V and 1W). At the age of 3 months, she presented with failure to thrive and vomiting and was found to be hypotonic. At 5 months of age, diffuse lung infiltrates were noted, developing to bronchial wall thickening and lung fibrosis on chest CT (Figures 1X and 1Y). Pads on dorsal phalanges and digital clubbing were observed. Histopathology revealed a combination of non-specific interstitial pneumonitis and desquamative interstitial pneumonitis with intra-alveolar cholesterol granuloma. The latter were surrounded by alveolar macrophages and T lymphocytes (Figures S1D–S1F). At age 10 years she has normal psychomotor development.

Participant 5 (P5, Figures 1A, 1AA, and 1AB) was the first child born to non-consanguineous parents. Their second child died from the consequences of extreme prematurity after delivery at 24 weeks gestation. Both parents had healthy children from prior partners. Participant 5 was a vaginal breech delivery at term with normal growth parameters but gained weight poorly with significant gastresophageal reflux. At 3 months of age, she was evaluated for marked hypotonia, and nerve conduction studies and a brain MRI showed no significant abnormality. She demonstrated gross motor delays, sitting at 10 months. At 2 years of age she was microcephalic with occipital frontal circumference below first percentile. She had distinctive facial features including deep-set eyes, a small nose, and full cheeks. A muscle biopsy was performed and showed normal histology with respiratory chain biochemistry consistent with a complex 1 deficiency, with normal results for complexes II–IV. She had continued difficulty gaining weight due to chronic vomiting and diarrhea, which were improved with pancreatic supplements. She was also found to have low serum albumin, hypertriglyceridemia, and proteinuria, leading to a renal biopsy showing features of focal segmental glomerulosclerosis. At age 2, she was also noted to have mild hepatosplenomegaly, with later liver biopsy showing chronic inflammation and macrovesicular steatosis. She gradually developed tachypnea and oxygen dependency by 3 years old. Pulmonary imaging and biopsy showed features of interstitial pneumonitis, which were believed to be secondary to aspiration, leading to fundoplication surgery. These measures improved feeding management and she was relatively stable at age 4, demonstrating normal speech and language and cognitive development. By age 6 her pulmonary status declined, and she was oxygen dependent 24 hr per day. Lung biopsy at age 8 revealed distortion of alveolar architecture, foreign body-associated giant cell reaction, numerous cholesterol clefts, and chronic inflammatory cell infiltrates. By 8 she required mechanical ventilation. She developed pulmonary hypertension and died suddenly at home of a cardiac arrest at 8 years.

No mutations in known disease genes explaining the phenotype were identified in any of the five affected participants. Exome sequencing in all five affected participants identified bi-allelic rare, predicted pathogenic variants in *FARSB* (GenBank: NM_005687.4) that were all confirmed by Sanger sequencing (Figure 1A). All missense variants occurred at highly conserved residues and were predicted to be pathogenic by multiple prediction algorithms, and CADD scores are shown in Table 1. All variants were either absent or present with a minor allele frequency of less than 1 in 1,000 in gnomAD. Participants 1 and 2 had paternally inherited canonical splice site variant, c.848+1G>A, and a maternally inherited missense variant c.914G>A (p.Arg305Gln), present at a highly conserved residue (Figure 1A). Participant 3 had a paternally inherited c.1202G>A (p.Arg401Gln) and a maternally inherited c.1381A>C (p.Thr461Pro) variant identified; participant 4 had a maternally inherited c.1202G>A (p.Arg401Gln) variant shared with P3 and a paternally inherited c.755T>C (p.Phe252Ser) identified; and participant 5 had c.784A>G (p.Lys262Glu) and c.226T>C (p.Cys76Arg) variants (Figure 1A).

In summary, all five individuals presented with common features of hypotonia and interstitial lung disease with cholesterol pneumonitis, all with bi-allelic variants in *FARSB*. Additional variable features were multisystemic and included the vasculature (extensive cerebral aneurysms, hypertension), brain (cerebral calcifications), liver (cirrhosis and transaminitis), intestines (malrotation), kidneys (proteinuria), connective tissue (scoliosis and pectus excavatum), and distinctive facial features.

### Prediction of Structural Impact of *FARSB* Missense Mutations

**Figure 2:**
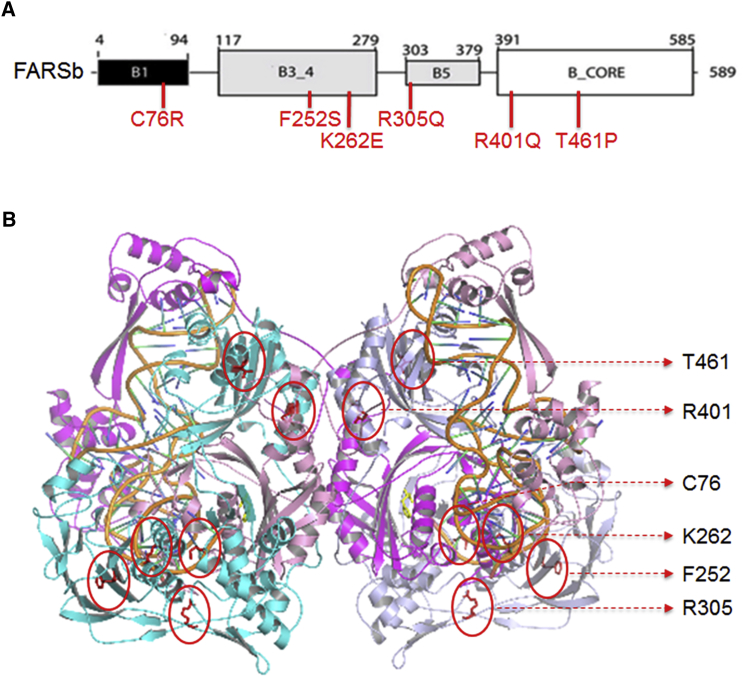
The Missense Mutations Are Located in Various Domains of FARSb (A) The schematic of FARSb protein and location of the missense mutations in various domains. (B) Location of the mutational sites on the structural model of the human FARS-tRNA^Phe^ complex. The protein and tRNA backbones are shown in cartoon representation. Two FARS α chains are colored in dark and light blue, two β-chains in dark and light magenta, and tRNA^Phe^ in orange. The amino acids at the mutational sites are shown in sticks and colored in red. Based on the model, residues Lys262 and Arg305 are closer to tRNA^Phe^ (<10 Å), while the other four residues (Cys76, Phe252, Arg401, and Thr461) have a longer distance (>15 Å) from tRNA^Phe^. None of these residues are directly involved in the interaction with tRNA.

**Figure 3:**
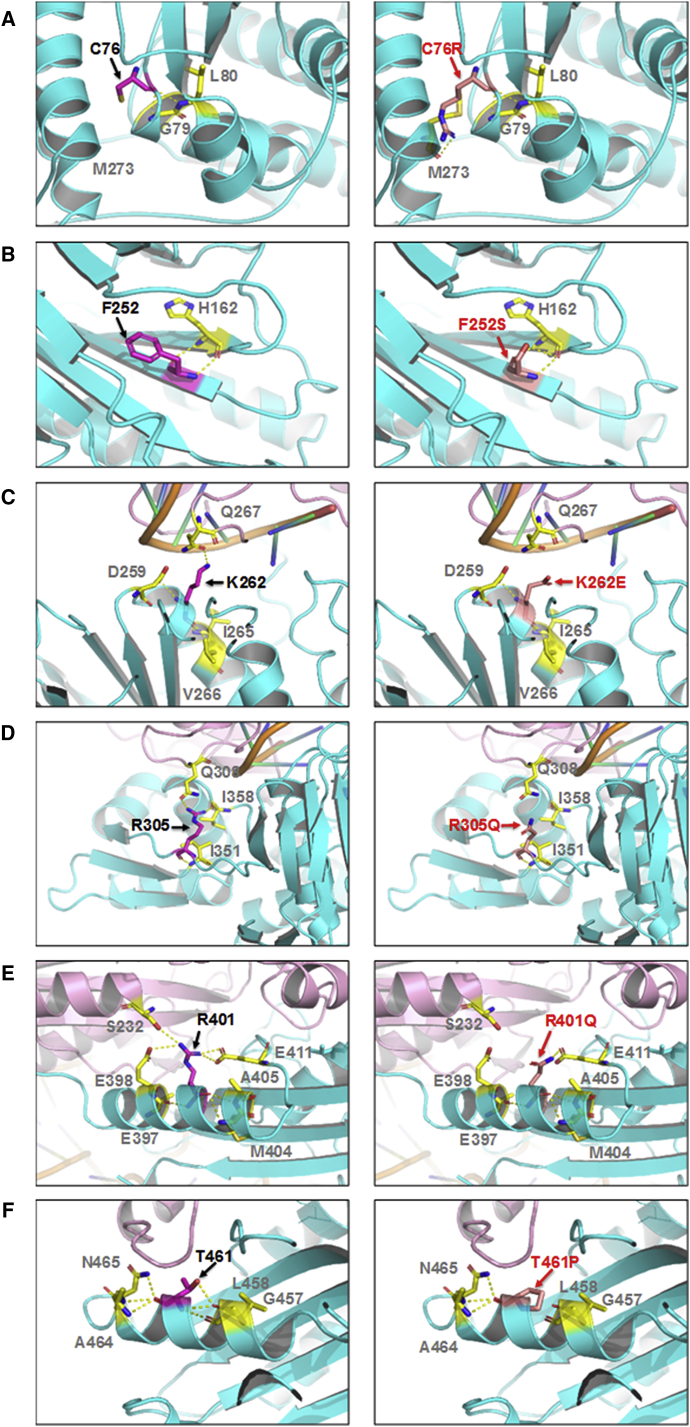
Predicted Impact of Missense Mutations on FARS Structure Pairwise comparisons between the wild-type and mutant residues for changes in local contacts with other amino acids. The highlighted residues are shown in sticks and labeled. The hydrogen bonds are presented as yellow dash lines. (A) Cys76 forms hydrogen-bonding with adjacent residues Gly79 and Leu80. The p.Cys76Arg mutation appears not to affect these hydrogen bonds but creates a new hydrogen bond with Met273 in the adjacent α-helix. (B) Phe252 forms hydrogen bonds with His162, which appear to be unaffected by the p.Phe252Ser mutation. (C) Lys262 is ∼6.5 Å from the tRNA CCA-end. It forms hydrogen bonds with adjacent residues Asp259, Ile265, and Val266 located in the same β-chain and Gln267 in the α chain. The p.Lys262Glu mutation is predicted to abolish the hydrogen bonding with α-Gln267. (D) Arg305 forms hydrogen bonds with residues Ile351 and Ile358 in the β-chain and Gln308 in the α-chain. The p.Arg305Gln mutation is predicted to disrupt the hydrogen bonding with β-Ile358 and α-Gln308. Arg305 has a distance of ∼8 Å to the tRNA^Phe^ and no direct contact. (E) Arg401 may form hydrogen bonds with Glu397, Glu398, Met404, Ala405, and Glu411 in the β-chain and Ser232 in the α-chain. The p.Arg401Gln mutation is predicted to disrupt the hydrogen bonding with α-Ser232. (F) Thr461 is located in an α-helical structure and forms hydrogen bonds with Gly457, Leu458, Ala464, and Asn465. p.Thr461Pro disrupts the hydrogen bonding with Gly457 and Leu458, and proline is known to be a helix-breaker, thus the p.Thr461Pro mutation is expected to disrupt the helical structure.

The six disease-associated missense mutations of *FARSB* are highly conserved and located in various domains including B1, B3_4, B5, and B-core (Figures 2A). The B3_4 domain is the editing domain of FARS. The B5 domain in the bacteria FARS was suggested to bind DNA.^45^, ^46^ The B-core domain dimerizes with the FARS α chain aminoacylation domain. The eukaryotic B1 domain is shorter than the bacterial counterpart, and its function is unclear. None of the mutated residues are known to be directly involved in aminoacylation, editing, or tRNA binding. To predict the impact of these mutations on FARS structure and function, we docked tRNA^Phe^ into the crystal structure of human FARS as described previously.^42^ We found that, in the FARS-tRNA^Phe^ model (Figure 2B), Lys262 has a distance of ∼6.5 Å to the 3′ end CCA tail of tRNA. In addition to the change of the residue charge from positive to negative, the p.Lys262Glu mutation is predicted to abolish the hydrogen bonding with Gln267 in the alpha chain (Figure 3C). This may lead to a change of local structure and affect loading of the amino acid to the tRNA CCA-end. The other residues have a longer distance from tRNA^Phe^ (∼8 Å for Arg305 and >15 Å for Cys76, Phe252, Arg401, and Thr461). In terms of the structural impact of the mutations, Thr461 is located in an alpha-helix, and its substitution by the helix breaker proline is expected to disrupt the helical structure, which possibly affects the overall protein structure (Figure 3F). The p.Cys76Arg mutation is predicted to create a new hydrogen bond with Met273 in the adjacent alpha-helix (Figure 3A). The residue charge changes from neutral to positive, and the extended side chain of arginine may introduce structural hindrance to affect the local structure. p.Arg305Gln and p.Arg401Gln are predicted to abolish some hydrogen bonding with spatially adjacent residues, and the change from positive to neutral charge may also eliminate electrostatic interactions (Figures 3D and 3E). These may affect the stability of local structures, but the mutational impact on the global structure and translational function of FARS is uncertain. Phe252 is located in the same β strand encompassing Glu254 and Thr256, the proposed key editing site on human FARS.^42^ p.Phe252Ser is predicted not to affect hydrogen bonding with adjacent residues, but this mutation removes the phenyl ring and changes the residue from hydrophobic to hydrophilic (Figure 3B). Whether this change affects the local structure and editing activity of FARS remains to be elucidated.

### The *FARSB* c.848+1G>A Mutation Alters Splicing and Acts as a Null Allele

**Figure 4:**
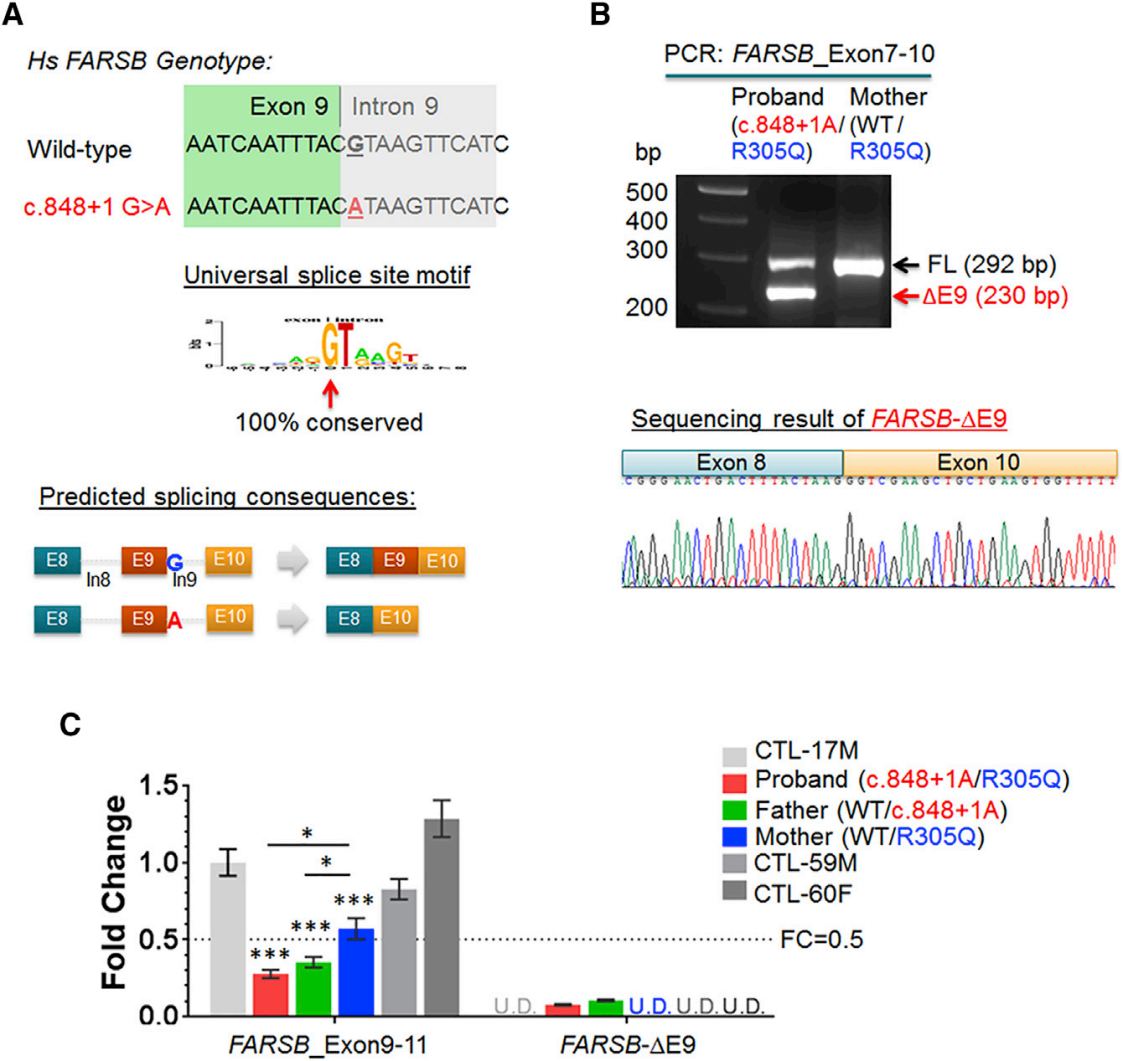
The c848+1G>A Intronic Mutation Resulted in Aberrant Splicing of *FARSB* (A) The mutation of the universal splice site motif is expected to affect the splicing of nearby exons in the *FARSB* gene, such as skipping of the adjacent exon 9. (B) A splice variant that deleted exon 9 of the *FARSB*_FL transcript, designated as *FARSB*-ΔE9, was detected in the proband but not in the mother. (C) Quantitative real-time PCR analysis of mRNA expressions of *FARSB*_E9-11 (using primers targeting exons 9 and 11 of *FARSB*, thus primarily detecting the full-length transcript), and *FARSB*-ΔE9 (using primers specifically amplifying the ΔE9 variant) in primary fibroblasts. Gene expression of *FARSB*_E9-11 and -ΔE9 in the fibroblasts of the proband, both parents, and three control subjects were calculated based on Ct values normalized to house-keeping genes *RPL9* and *RPS11*. The fold changes were thus calculated by relative to the *FARSB*_E9-11 level of CTL-17M. Data were presented as mean ± SEM. The ΔE9 RNA was detected only in the proband and father cells but not in the mother and control subjects (U.D. denotes no detectable amplification within 45 qPCR cycles).

The c.848+1G>A substitution of P1 and P2 alters a canonical splice site^47^ and is predicted to result in skipping of exon 9 and then to cause a frameshift (Figure 4A). We performed PCR on cDNA prepared from mRNA extracted from whole blood and used primers flanking exons 7 and 10 to analyze transcripts that should include exon 9. As expected, the *FARSB* transcript with deletion of exon 9, *FARSB*-ΔE9, was detected in the proband who carries the c.848+1G>A mutation (Figure 4B). In contrast, the mother who does not carry this splice mutation expressed only the full-length transcript. Disruption of canonical splicing of intron 9 reduced the amount of full-length transcript in the proband compared to the mother. Next, using quantitative real-time PCR, we quantified the expression of *FARSB* in primary fibroblasts from the proband, both parents, and three normal control subjects (Table S1). Consistent with the above PCR results, *FARSB*-ΔE9 expression was detected only in the proband and the father who both carry the c.848+1G>A mutation, and not in the mother or in the three control subjects (Figure 4C). Relative to the mother and controls, there was a reduction of more than 50% of the full-length *FARSB* transcript for the proband and father (to 27.5% and 35.2% of control, respectively).

Exon 9 is located in the middle of the *FARSB* transcript and encodes the end of the B3-4 domain and partial linker to the B5 domain (Figure S2A). Failure to include exon 9 with 62 nucleotides changes the reading frame and is predicted to introduce a premature termination codon in exon 10 (after joining of exons 8 and 10) and is predicted to activate nonsense-mediated mRNA decay.^48^ Indeed, western blots using an N-terminal FARSb antibody targeting the region of amino acids 161–248 showed no protein product of the ΔE9 variant in the primary fibroblasts or immortalized peripheral blood mononuclear cells (PBMCs) (Figure S2B and Table S1). These results are consistent with the expected consequences of the canonical splice c.848+1G>A substitution in *FARSB* and suggest that this allele acts as a loss of function.

**Figure 5:**
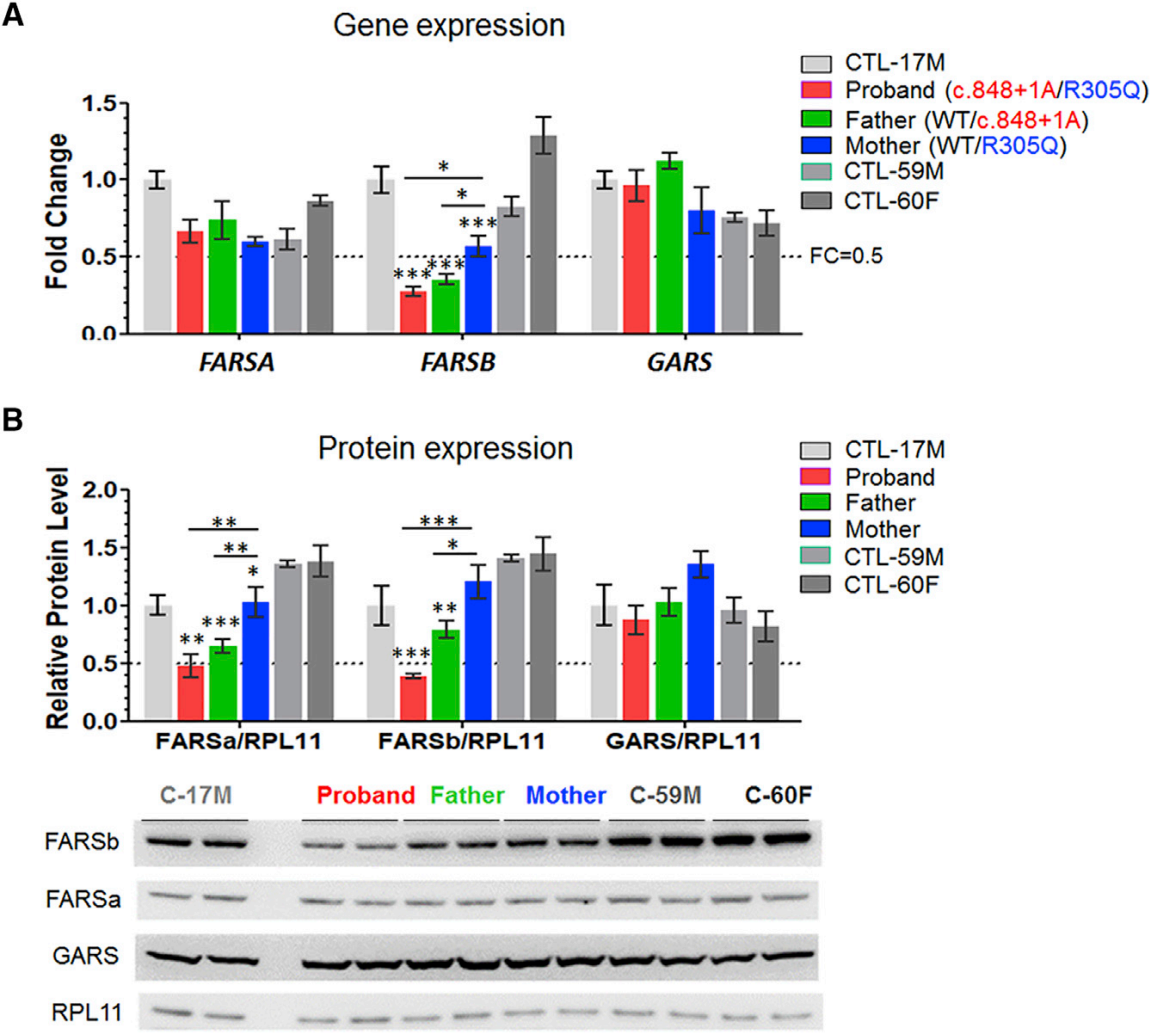
Compound Heterozygous *FARSB* Mutations Reduced FARS Protein Level in Proband Fibroblasts (A) Gene expression of *FARSA*, *FARSB*, and *GARS* in primary fibroblasts from proband, parents, and control subjects by qPCR analysis relative to house-keeping genes *RPL9* and *RPS11* (n = 3). (B) Representative western blot results (bottom panel) and calculated relative protein levels (upper panel, n = 4) of FARSa, FARSb, and GARS in primary fibroblasts. RPL11 was employed as the loading control. Data were presented as mean ± SEM. Significant difference compared to the respective age- and sex-matched control is indicated by asterisks and labeled directly above the sample bars, and that between any two cells of the proband family is indicated by asterisks above the lines (^∗^p < 0.05, ^∗∗^p < 0.01, and ^∗∗∗^p < 0.001 by one-way ANOVA followed with Newman-Keuls’ multiple comparisons).

We found no differences in transcript levels of *FARSA* in fibroblasts among the six cell lines (affected individual, parents, or three normal control subjects) (Figure 5A).

### Comparing Transcript and Protein Levels of FARS α- and β-Subunits

To further characterize the impact of the recessive mutations, we compared transcripts and protein levels of *FARSB* in fibroblasts from the proband, parents, and three age-matched unrelated control subjects (Figure 5). FARS α-subunit (FARSa) and cytoplasmic glycyl-tRNA synthetase (GARS) were evaluated in parallel and, in all six cases, expression of GARS transcripts and protein were identical. Similar expression of α-subunit transcripts was observed for all six subjects, but the amount of α-subunit protein was substantially reduced for the proband and father. Because the proband and father clearly had decreased amounts of β-chain protein (FARSb), the results suggest that the β-chain was needed to stabilize the α-chain. Similar results were observed in immortalized PBMCs (Figure S2C). The compound heterozygous mutations were associated with ∼80% reduction of protein levels of both the FARSb and FARSa.

### Protein Synthesis Is Not Impaired in Proband Cells

For aminoacylation analysis, we used whole-cell extracts from immortalized PBMCs normalized for total protein content. We measured charging of Phe to yeast tRNA for one normal control subject, the father, mother, and proband. Aminoacylation rates of the father and mother were 67%–75% of the control subject, while the proband was 37% of the control subject (Figure S3A). This apparent reduction in aminoacylation rates was correlated with and accounted for by a reduction of FARS protein levels in the proband, father, and mother relative to the control (19%, 36%, and 55% of the control, respectively; Figure S2C). Both the GARS (an internal control) protein levels and rates of GARS aminoacylation for all four samples were similar (Figure S3B). Thus, the p.Arg305Gln mutation has little effect on charging activity in immortalized PBMCs and, based on other work,^19^, ^49^ suggests that decreased levels of FARS activity in the proband are unlikely to affect protein synthesis. To further evaluate this observation, we used a puromycin incorporation assay to investigate protein synthesis. We found that the affected individual’s PBMCs had similar rates of protein synthesis as those from a control subject and father (Figures S3C–S3E).

**Figure 6:**
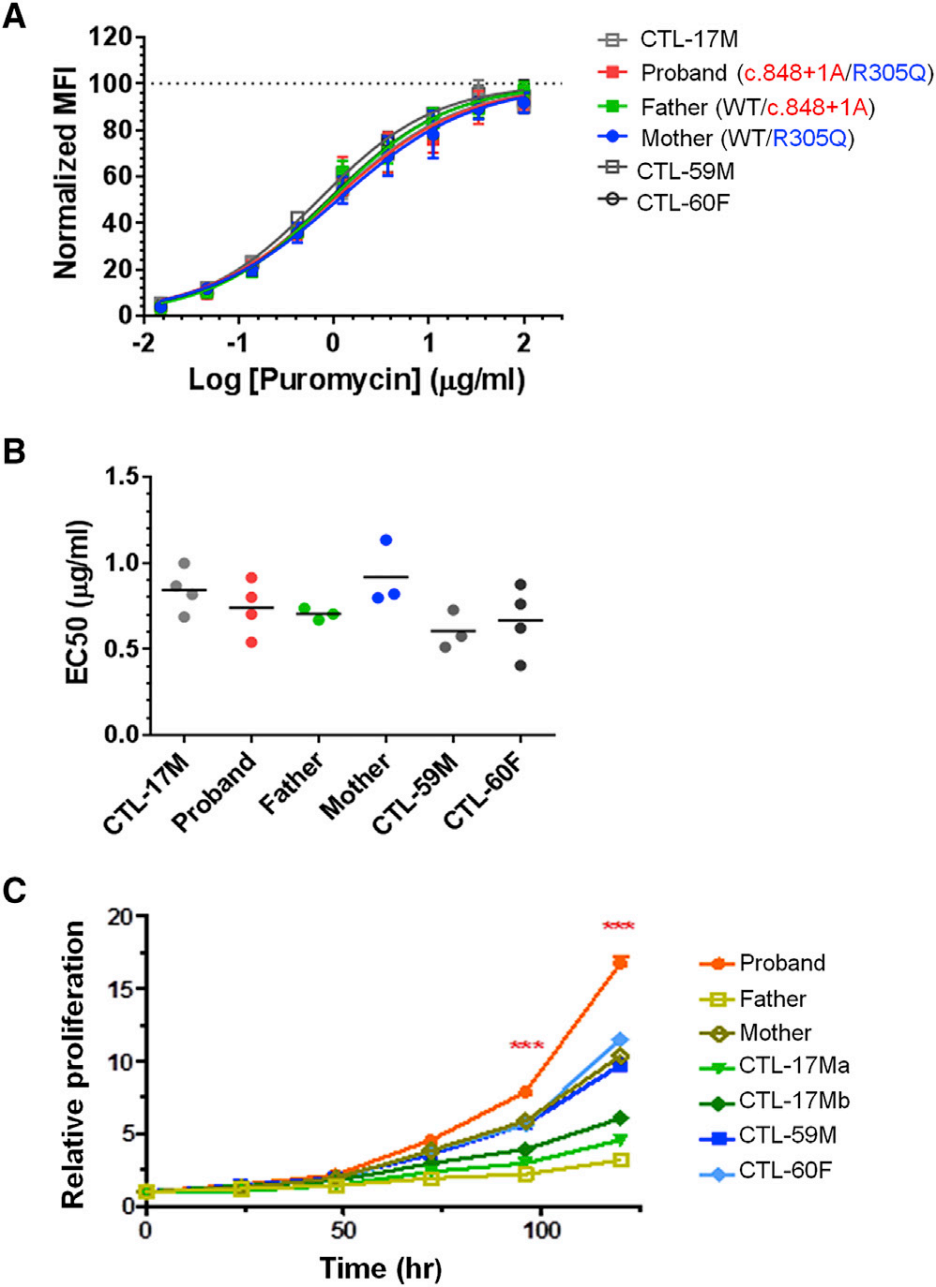
Protein Synthesis Was Not Impaired in the Proband Fibroblasts (A) The global protein synthesis was evaluated by puromycin incorporation in cultured fibroblasts of the proband, family, and control subjects. Shown are mean ± SEM of normalized median fluorescence intensity (MFI) from 3–4 separate experiments (left panel). The dose-response curves were fitted by log(agonist) versus normalized response − variable slope nonlinear regression (Prism). (B) Dot-plot of the calculated EC_50_ of puromycin incorporation (each independent test shown as a dot). No statistical significance was found among the groups based on the one-way ANOVA (p > 0.15). (C) Cell proliferation of cultured fibroblasts of the proband, both parents, and four control subjects. Data were presented as mean ± SEM. Time courses revealed faster proliferation in the proband fibroblast than others (^∗∗∗^p < 0.001).

We measured rates of protein synthesis in primary fibroblasts from the proband, parents, and three normal subjects, using the puromycin incorporation assay (Figure 6A). A dot plot of the results from 3–4 independent replicates of each of the 6 independently sourced fibroblasts showed no significant difference in rates of protein synthesis (p > 0.15; Figure 6B). Thus, with these primary cells we also did not observe a significant loss of the capacity for protein synthesis.

To confirm that protein synthesis was sufficient to support cell growth, primary fibroblast cell proliferation rates were measured. Interestingly, primary cell proliferation was significantly more rapid in the proband compared to three control subjects (p < 0.001) (Figure 6C). These data further support the conclusion that any reduced level of FARS is not affecting the capacity for protein synthesis in the affected individual.

## Discussion

We describe a novel genetic disorder with an unusual multi-organ phenotype of interstitial lung disease with cholesterol pneumonitis, intracranial aneurysms, cerebral calcifications, hypotonia, and liver cirrhosis caused by bi-allelic mutations in *FARSB*. Other less consistent features include renal disease, intestinal malrotation, and dysmorphic facial features. The most consistent and life-limiting feature so far has been pulmonary disease although participants were not systematically investigated for all the features observed across all individuals, and were evaluated at different ages. The associated mutations include a c.848+1G>A splice mutation that leads to exon skipping and a frameshift in the transcript, and decreased transcript and protein levels of *FARSB*, which collectively suggest that this is a loss-of-function allele. Both FARSb and FARSa protein levels were diminished, demonstrating the deleterious effects of these mutations on FARSb protein levels and, as a consequence, the destabilization of FARSa. The missense p.Arg305Gln allele is associated with slightly reduced aminoacylation rates that correlated with the reduced amount of FARS protein. Yet, the proband had normal rates of protein synthesis and increased cellular proliferation in the investigated cells. We also identified three additional, unrelated individuals with overlapping phenotypes, each with compound heterozygous missense mutations in *FARSB.* The lack of any phenotype in carrier parents and carrier siblings suggest that only the individuals with the compound heterozygous mutations have fallen below a critical threshold for a secondary function of *FARSB* beyond that of protein translation. Recently, another family with an affected child with bi-allelic, *FARSB* variants (p.Thr256Met and p.His496Lysfs^∗^14) that includes one predicted loss-of-function allele and a missense variant with very similar clinical features was identified,^50^ supporting our conclusions.

In previous efforts to investigate the impact of aaRS genetic mutations on the canonical enzymatic function, the *in vitro* aminoacylation assays were commonly employed to study enzyme kinetics.^16^ Some studies also measured the amount of charged tRNA in participants’ cells or analyzed the mutated genes in yeast complementation assays.^23^, ^24^, ^25^, ^30^, ^51^ However, it is unclear how well the changes observed in these assays correlate with a significant compromise of cellular translation. The puromycin incorporation assay used here is an effective way to evaluate overall protein synthesis in cultured cells, to test whether a mutation affects the cellular translational machinery and, if not, to point to a disease mechanism due to an orthogonal, non-canonical function of aaRS. Consistently, we demonstrated that the proband’s cells harboring the bi-allelic *FARSB* mutations showed no decline of overall protein synthesis.

There are a total of 37 human aaRSs (17 cytoplasmic, 17 mitochondrial, and 3 bifunctional), each of which is specific for a single amino acid.^16^ These enzymes are highly conserved and ubiquitously expressed across all tissues. The aaRSs also have roles outside of protein synthesis, some of which are independent of this catalytic activity and include nuclear regulation of transcription, extracellular receptor mediated signaling, and mTOR regulation.^14^

Most aaRSs that have been implicated in human diseases are associated with primary manifestations in the central and peripheral nervous system. To date, 31 of the 37 aaRSs have been linked to monogenic diseases.^16^ Given the essential and non-redundant functions of these enzymes, recessively inherited aaRS disorders are generally caused by compound heterozygous hypomorphic alleles rather than by homozygous null alleles.^16^

Although the novel disease caused by *FARSB* mutations described here shares some similarities with other known aaRS disorders, it is unique in other ways. None of the known aaRS diseases have extensive vascular and multi-organ manifestations, involving the lungs, brain, liver, kidney, intestine, and vasculature. However, other diseases caused by mutations encoding both non-polar and hydrophobic amino acid aaRSs share some clinical aspects with our *FARSB* individuals. For instance, recessive mutations in *MARS* (encoding methionyl-tRNA synthetase) (MIM: 615486) are associated with hypotonia, endocrine dysfunction, liver disease characterized by lobular disarray, canalicular cholestasis, steatosis, and iron deposition.^25^ Of interest, individuals with *MARS* mutations share with all five participants described here an extremely rare interstitial lung disease defined by cholesterol pneumonitis.^32^, ^52^ Autosomal-recessive *IARS* (encoding isoleucyl-tRNA synthetase) (MIM: 617093) deficiency is associated with liver disease, hypotonia, and intellectual disability.^23^, ^24^ In contrast to our findings with deficiency of cytoplasmic *FARSB*, compound heterozygous mutations in nuclear-encoded *FARS2* (the mitochondrial phenylalanyl-tRNA synthetase) lead to combined oxidative phosphorylation deficiency associated with global developmental delay, refractory seizures, and lactic acidosis.^53^ Interestingly, in three individuals (P1, P3, and P5) a mitochondrial disease was suspected clinically. In P5, complex I activity was decreased, an observation similar to individuals with mutations in *IARS*.^23^ FARS, MARS, and IARS are part of a multi-synthetase complex (MSC), which is organized by nine cytoplasmic aaRSs and three aaRS-interacting multifunctional proteins AIMP1, AIMP2, and AIMP3.^2^, ^3^, ^54^ Some aspects of the clinical phenotype may be related to such non-canonical functions or to involvement in the MSC.

Thus, despite the essential role of aaRSs in protein translation in all cells, recessive mutations in aaRSs lead to tissue-specific disease phenotypes that are likely due to the specificity of the additional role of these transcripts in other tissue-specific cellular functions beyond protein translation. Our results define a new genetic syndrome characterized by diffuse parenchymal (interstitial) lung disease, hypertension, intracranial aneurysms, cerebral calcifications, and liver cirrhosis due to compound heterozygous mutations in *FARSB.* These findings expand our knowledge of the diverse roles of aaRSs in human disease and further support the importance of the now well-established expanded functions of the higher eukaryote tRNA synthetases, some of which (e.g., DNA binding^45^, ^46^) may have their roots in bacterial ancestors. Further functional assays of these *FARSB* variants in modeling systems are in progress and will cast light on the disease mechanism and the expanded functions of FARS.

## Declaration of Interests

Z.X., Y.E.C., L.A.N., X.-L.Y., and P.S. have a financial interest in aTyr Pharma, although none specifically in this work.

## Accession Numbers

The accession numbers for the sequences reported in this paper are ClinVar: SUB4204969, SUB4204245, SUB4187351, SUB4090058, SUB4090045, SCV000778460, and SCV000778461.

## Web Resources

Align GVGD, http://agvgd.hci.utah.edu/agvgd_input.php
CADD, http://cadd.gs.washington.edu/
ClinVar, https://www.ncbi.nlm.nih.gov/clinvar/
GenBank, https://www.ncbi.nlm.nih.gov/genbank/
gnomAD Browser, http://gnomad.broadinstitute.org/
OMIM, http://www.omim.org/
PolyPhen-2, http://genetics.bwh.harvard.edu/pph2/
PyMOL, https://pymol.org/2
RCSB Protein Data Bank, http://www.rcsb.org/pdb/home/home.do
Revel, https://omictools.com/rare-exome-variant-ensemble-learner-tool
SIFT, http://sift.bii.a-star.edu.sg/

## Supplemental Data

Document S1. Figures S1–S3 and Table S1

Document S2. Article plus Supplemental Data
