# Supplementary material for: Bi-allelic Mutations in Phe-tRNA Synthetase Associated with a Multi-system Pulmonary Disease Support Non-translational Function
Source: Am J Hum Genet. 2018 Jul 5;103(1):100–14. doi: 10.1016/j.ajhg.2018.06.006 (PMC6035289; doi:10.1016/j.ajhg.2018.06.006)
Supplement: Document S1. Figures S1–S3 and Table S1 [file mmc1.pdf]

The American Journal of Human Genetics, Volume *103*

Supplemental Data

Bi-allelic Mutations in Phe-tRNA Synthetase Associated with a Multi-system Pulmonary Disease Support Non-translational Function

Zhiwen Xu, Wing-Sze Lo, David B. Beck, Luise A. Schuch, Monika Oláhová, Robert Kopajtich, Yeeting E. Chong, Charlotte L. Alston, Elias Seidl, Liting Zhai, Ching-Fun Lau, Donna Timchak, Charles A. LeDuc, Alain C. Borczuk, Andrew F. Teich, Jane Juusola, Christina Sofeso, Christoph Müller, Germaine Pierre, Tom Hilliard, Peter D. Turnpenny, Matias Wagner, Matthias Kappler, Frank Brasch, John Paul Bouffard, Leslie A. Nangle, Xiang-Lei Yang, Mingjie Zhang, Robert W. Taylor, Holger Prokisch, Matthias Griese, Wendy K. Chung, and Paul Schimmel

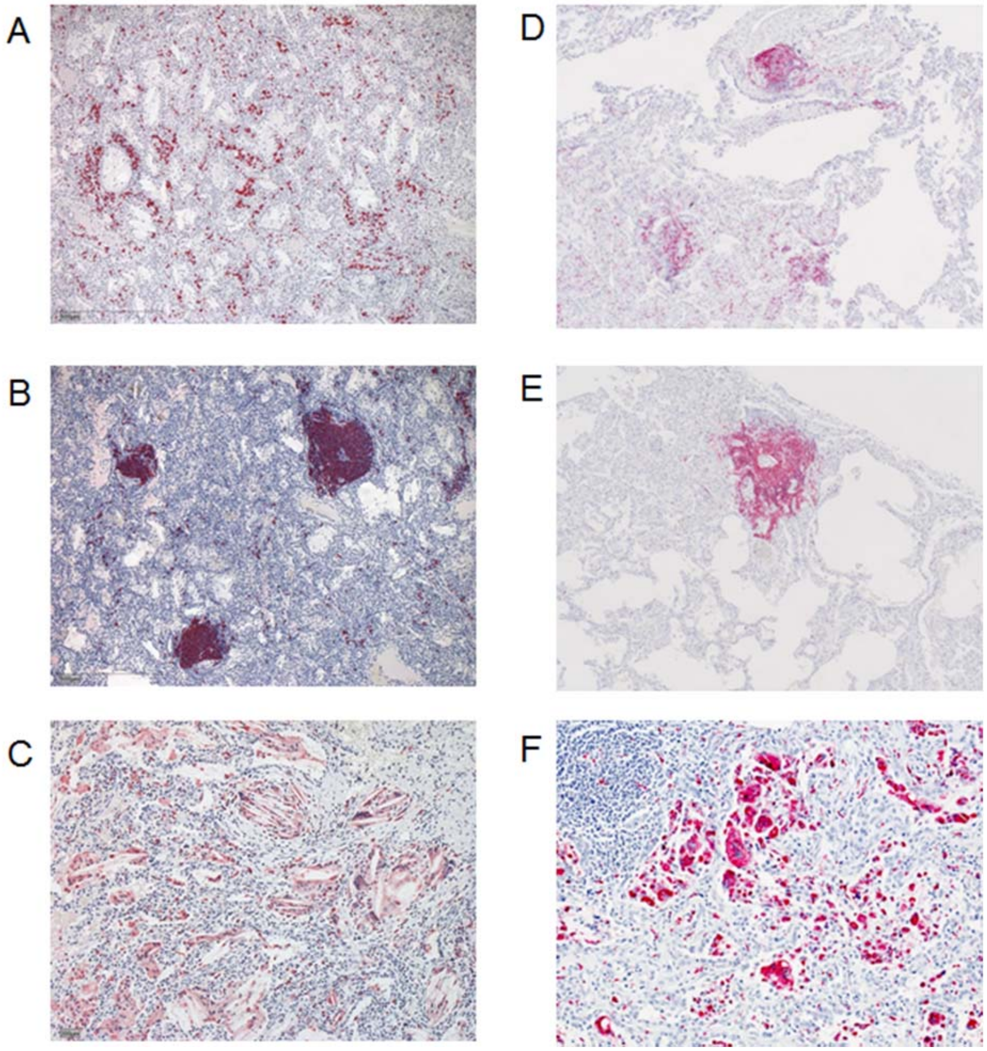

Figure S1. Lymphocytes and alveolar macrophages in the lungs. Staining with antibodies against CD3 shows T-lymphocytes (A, D), against CD20 B-lymphocytes (B, E), and against CD68 alveolar macrophages (C, F). Note accumulation of T cells around cholesterol crystals, B cells in follicles, and macrophages in the alveolar space as usually and in and around cholesterol granulomas (F). Participant P3 (A-C), participant P4 (D-F).

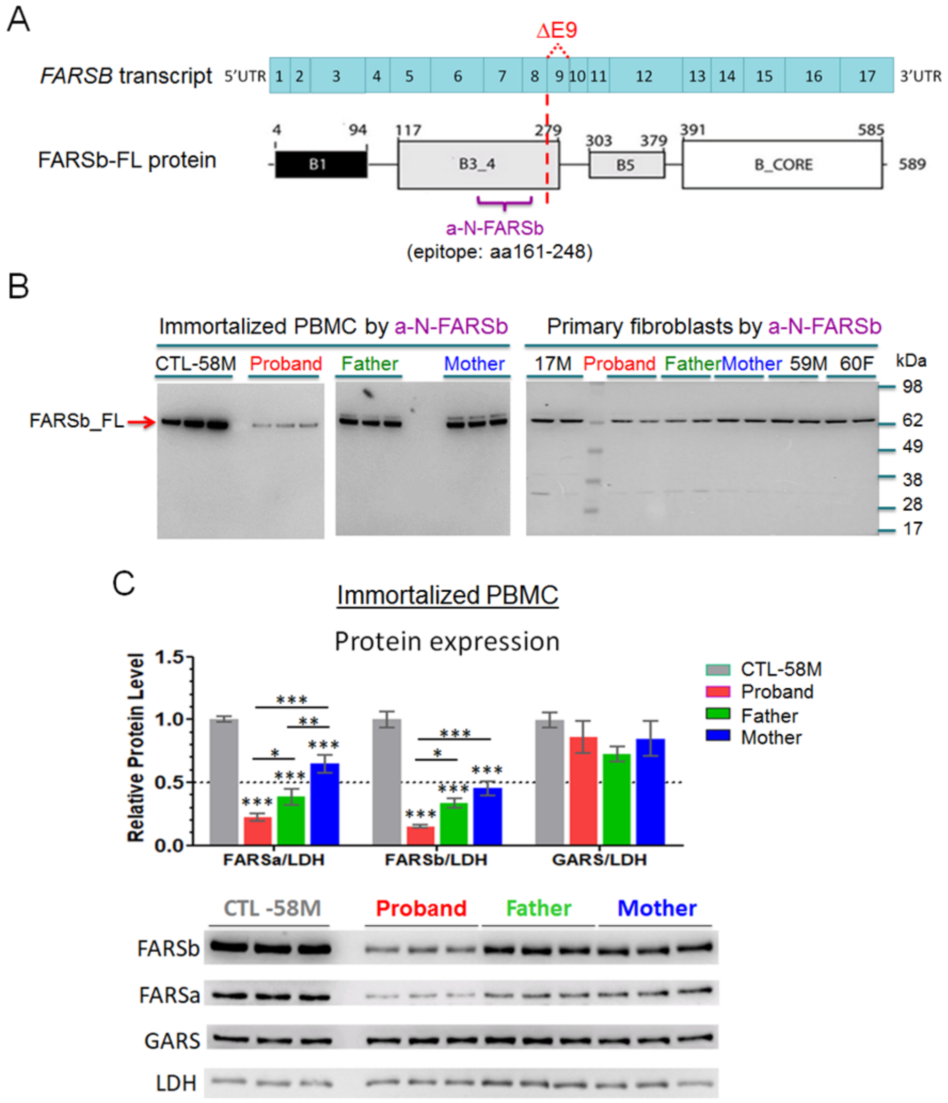

Figure S2. Effect of *FARSB* mutations on FARS protein levels. (A) Schematic illustrations of the *FARSB* transcript (exons in scale with the length), skipping of exon 9 (ΔE9), and FARSb-FL protein (aligned with the encoding exons). The FARSb-FL protein is composed of B1, B3_4, B5 and B_core domains. The B3_4 domain is the editing domain and the B-core domain dimerizes with the α-subunit aminoacylation domain. (B) Western blot of total cell lysates of immortalized PBMCs and primary fibroblasts using the anti-N-FARSb (targeting aa161-248). No ΔE9-like band was detected in both cell types. Although a protein band with the size between 28 and 38 kDa was observed in primary fibroblasts, it was also present in mother and control cells indicating that it was unlikely to be the protein product of ΔE9. (C) Shown are representative western blot results and calculated relative protein levels (n = 6) of FARSa, FARSb and GARS in immortalized PBMCs. LDH was employed as the loading control. Significant differences compared to the CTL-58M control is indicated by asterisks and labeled directly above the sample bars, and that between cells of any two individuals is indicated by asterisks above the lines (*p < 0.05, **p < 0.01 and ***p < 0.001).

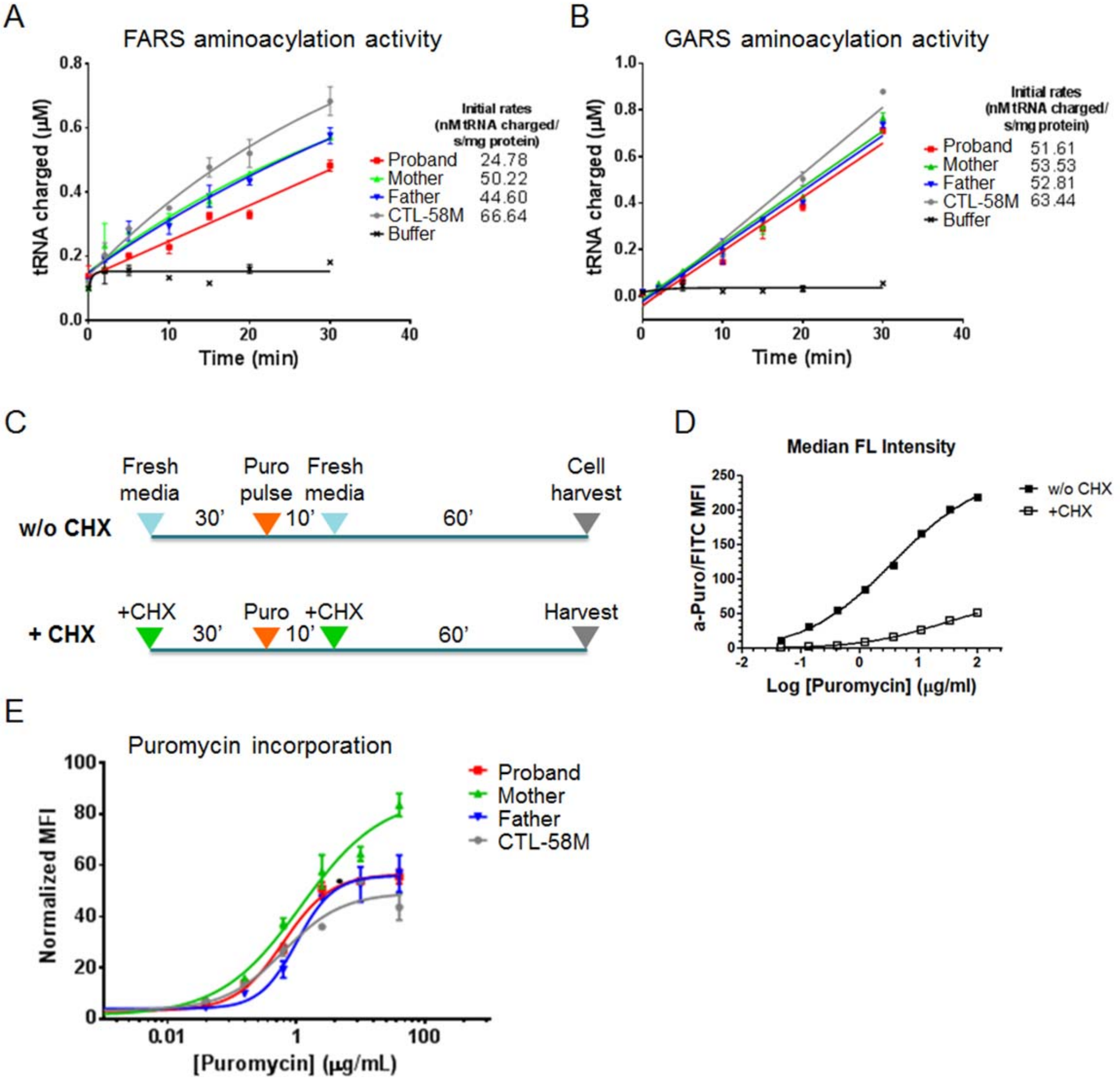

Figure S3. Aminoacylation and protein synthesis rates. (A) FARS aminoacylation activity of cell lysates from immortalized PBMCs. (B) GARS aminoacylation activity of cell lysates from immortalized PBMCs. (C) Illustration of treatment protocols of puromycin (Puro) impulse in the absence (w/o) or presence of cycloheximide (+CHX). Cells were seeded one day before treatment and changed with fresh media ± CHX. After 30 mins’ incubation, cells were pulsed with Puro for 10 min, which was removed by a media refresh. Cells were incubated for another 1 hour to allow puromycin incorporation, and harvested for subsequent flow cytometry analysis. (D) Representative puromycin incorporation curves of proband primary fibroblasts with (open circles) or without (closed circles) CHX inhibition of protein synthesis. (E) Puromycin incorporation rates of cultured immortalized PBMCs.

**Table S1. Information of cells employed in this study**			
**Cell type**	**Cell name**	**Source (Cat#)**	***FARSB* genotype**
Immortalized PBMC[a]	CTL-58M	ATCC (#CRL5959)	wildtype
	Participant 1	Participant derived	c.848+1 G>A, R305Q
	Father	Participant derived	c.848+1 G>A
	Mother	Participant derived	p.R305Q
Primary fibroblasts	CTL-17Ma	Coriell (#GM07753)	wildtype
	CTL-17Mb	Coriell (#GM07492)	wildtype
	CTL-59M	Participant	wildtype
	CTL-60F	Participant	wildtype
	Participant 1	Participant	c.848+1 G>A, R305Q
	Father	Participant	c.848+1 G>A
	Mother	Participant	R305Q

[a]We first obtained the immortalized PBMCs and analyzed these cells (results are presented in Supplementary Data). Later we obtained the primary fibroblasts and preformed similar experiments with these primary cells (results are mostly presented in main figures). In general, results from these two types of cells are highly consistent.
